# Effect of Opioids on Survival in Patients with Cancer

**DOI:** 10.3390/cancers14225720

**Published:** 2022-11-21

**Authors:** Jason W. Boland

**Affiliations:** Wolfson Palliative Care Research Centre, Hull York Medical School, University of Hull, Hull HU6 7RX, UK; jason.boland@hyms.ac.uk

**Keywords:** opioid, survival, neoplasms, cancer, immune, pain, oncology, palliative care

## Abstract

**Simple Summary:**

Opioids, such as morphine, are strong pain killing medicines. Patients with cancer often need opioids to manage their pain. Although they might be helpful for pain control, opioids have side effects. Some of these side effects are obvious such as constipation and confusion. Other side effects are less overt, such as effects on the immune system and other effects on cancer growth. There have been studies in patients with advanced cancer which show that patients taking opioids have a shorter survival than patients not taking opioids. There are other differences in these groups of patients which might also account for the differences in survival. These include the possibility that patients needing opioids might have more aggressive cancers which cause more pain, which is why this group of patients take more opioids. It is also possible that the more aggressive cancer is what shortens survival. Future studies are needed to understand the cause of the effects of the underlying cancer type, pain, specific opioids, and survival. Until this is better understood, opioids should continue to be used to control pain in patients with cancer.

**Abstract:**

Opioids are commonly used for pain management in patients with cancer. They have a range of unwanted effects, including some that potentially influence cancer growth. This article reviews the data assessing the effects of opioids on survival in patients with cancer. Many studies assessing this show an association between opioids and decreased survival. This effect is present even at very low doses of opioids. These studies do not assess causality, so it is not known if it is a direct effect of opioids on survival. As the control groups are not matched to the opioid group it might be that opioids are being used to control pain and patients receiving opioids have more aggressive cancers and it is the underlying cancer which is causing the decreased survival. Furthermore, although some studies allude to different opioids having different effects on survival, often all opioids are pooled in analysis. Future work needs to try to ascertain causality and differentiate between different opioids, pain, and cancer-mediated effects on survival in specific cancer types. Until then, opioids should continue to be used in patients with cancer as part of measures to optimise comfort and quality of life.

## 1. Introduction

Patients with cancer often develop symptoms for which opioids are prescribed to help improve comfort and quality of life [1,2]. In patients with cancer opioids are often used to manage pain, as well as shortness of breath, they are also sometimes used in the management of diarrhoea and cough. Commonly used opioids include morphine, oxycodone, codeine, tramadol, fentanyl, buprenorphine, hydromorphone, and methadone [1,3,4]. Although all opioids bind to and have effects at the opioid receptors, they have variable interactions with the different opioid receptors and actions on a range of different non-opioid receptors [5]. These different receptor binding profiles of opioids lead to different clinical effect profiles for this diverse group of drugs, with different prevalence of adverse effects for different opioids [1,6]. Many of these effects are overt such as pain reduction, constipation and confusion (Table 1) [1,7,8]. Some effects are less clear immediately, such as those on the immune and endocrine systems (Table 1) [1,6,9]. The immune and other opioid effects might influence survival [10,11,12].

The administration of opioids differs between settings and indications in patients with cancer pain. They might be used in the short term, such as peri-operatively for the resection of cancer, or in the longer term, often in patients with ongoing cancer pain. Often for shortness of breath lower doses are advocated compared with what some patients need for cancer pain management [13,14]. Even so, some patients only need low dose intermittent opioids to manage their cancer pain, whereas other need high doses of regular opioids. All these administration differences impact on the effects of opioids. This includes different immune effects of opioids and their clinical consequences. As patients undergoing cancer-resection surgery have many different variables compared to patients with ongoing cancer related pain, including often short-term peri-operatively opioids, anaesthesia, and surgery-related immune changes [15,16], the effects of peri-operative opioids on survival are out with the scope of article. This article will focus on opioids in patients with moderate to severe ongoing cancer related pain.

## 2. Clinical Concerns about the Effect of Opioids on Survival in Cancer

Data have emerged highlighting that opioids might be associated with a decreased survival [11,12]. The effect of opioids on survival might be variable in circumstances different than opioids being used proportionately in patients with cancer. Such circumstances include opioids used recreationally outside the clinical setting, opioids used by people with addiction, accidental and deliberate overdose. Opioids might also be used inappropriately in the clinical setting, especially if patients are not properly assessed and the opioid dose is titrated based on incomplete clinical review with lack of opioid knowledge. However, these concerns based on harms from opioid use in different settings have been extrapolated by some into the clinical setting in patients receiving opioids appropriately for cancer pain. This fear of opioids being associated with decreased survival, in different settings, might lead to opiophobia, among patients and clinicians, resulting in decreased prescription of opioids for cancer pain and decreased concordance.

It is also not uncommon for patients to not want opioids as they have seen opioids used for symptom control in dying patients and that patient has died on opioids. Although the opioids might have been appropriately used to control pain at the end of life, sometimes people focus on the association of opioids being administered and the patient dying, and not taking into account that the patient would have died anyway, possibly in more discomfort. This could be considered a bystander effect of opioids being used appropriately to control symptoms in patients who are dying. This association is often reported by people who have seen someone dying soon after a syringe driver (continuous subcutaneous infusion) being started to control symptoms. If appropriate doses are used, it is not the opioid in the syringe driver causing the death. The syringe driver is started due to a clinical deterioration, meaning the dying patient can no longer take opioids orally. The patient continues to deteriorate and die as expected, with the syringe driver in place to increase comfort. As the death occurs soon after the syringe driver started, the association might be thought to be causative. Explanation is essential prior to and during commencement of the syringe driver. It can take time and understanding to enable to people to untangle these notions. There are thus many reasons why patients, family and clinicians might think opioids shorten survival, especially in the non-clinical setting or when used inappropriately. It is important we are aware of the clinical data in the appropriate setting, assessing the effects of opioids on survival in patients with cancer pain [10].

Before looking at the clinical data assessing the effects of opioids on survival in patients with cancer in the appropriate setting, some of the mechanisms will be overviewed. These include the opioid anti-cancer immune interactions, non-immune effects of opioids which might impact on survival. The effect of tumour mu-opioid receptor (MOR) expression and cancer progression will be summarised. Hazard ratios (HR) compare the rate of events in a treatment group and the rate of events in a control group. In the studies included below, HRs depict the change in risk of death between groups of patients on high dose opioids and low dose/no opioids. The higher the HR is above 1, the more likely the risk of death from the intervention (a HR of 2, indicates twice the risk of death in the intervention group). In the studies included in this article, the HRs included are those which have been adjusted for other factors which can be corrected for, which might affect prognosis.

## 3. Mechanisms How Opioids Might Affect Survival in Patients with Cancer

**Figure 1:**
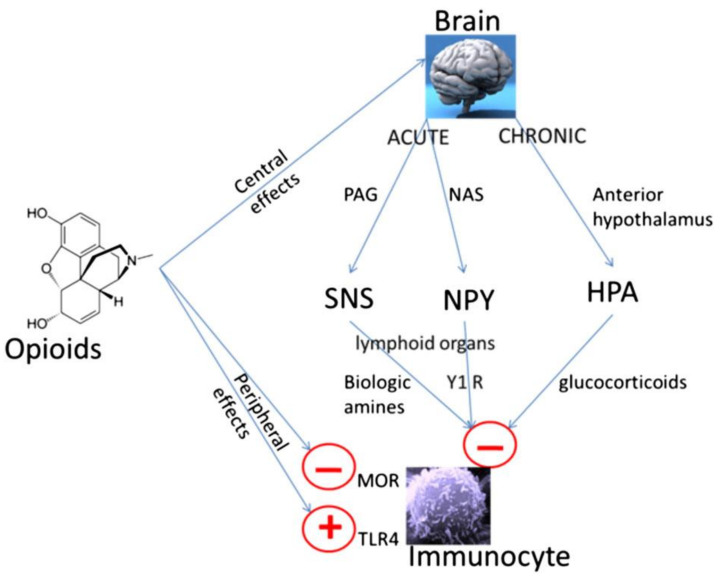
**Peripheral and central mechanisms of opioid-induced immune suppression.** Opioids can have direct effects on immune cells which express appropriate receptors such as the mu opioid receptor (MOR) and Toll-like receptor-4 (TLR-4); although there are differences between opioids [6,21,23,24]. Opioids can also have centrally mediated immunosuppressive effects [26,27]. Immediate central effects of opioids include enhancement of periaqueductal gray (PAG) activity. This in turn causes activation of the sympathetic nervous system (SNS). By innervating lymphoid organs, activation of the SNS leads to the release of biological amines which decreases cytotoxicity of NK cells and splenic lymphocyte proliferation [26]. In rodent models, morphine has acute effects via D1 dopamine receptors in the nucleus accumbens shell, which leads to an increase in the release of neuropeptide Y (NPY), this then reduces splenic NK cell cytotoxicity [27]. Chronic opioid administration increases activity in the hypothalamic pituitary adrenal (HPA) axis leading to glucocorticoid production, decreasing cytotoxicity of NK cells [26]. Reproduced with permission by Boland, J.W. et al. *Br. J. Cancer* **2014**, *111*, 866–873. [22].

There are a range of studies looking at how the effects of opioids might affect survival in patients with cancer [17,18,19,20]. These include studies looking at the diverse range of effects opioids have, such as cancer-related immunological effects of opioids, and how that impacts on survival [15,21]. Opioid-immune effects can be due to direct actions of opioids on immune cells (via MOR and Toll-like receptor 4; TLR4) or mediated centrally (via the sympathetic nervous system and hypothalamic pituitary adrenal axis) (Figure 1) [15,22]. Toll-like receptor 4 (TLR4) is an innate immune receptor which is activated by lipopolysaccharide (LPS), from the cell wall of Gram-negative bacteria [23,24]. This results in a pro-inflammatory response, which can be modulated by opioids [24]. The effect of opioids via TLR4 varies dependent on the cell type and activating stimulus. There is also TLR4/opioid receptor pathway crosstalk at multiple levels; this crosstalk can be different depending on the activating stimulus and cell type [23]. Opioids can be pro-inflammatory (activating LPS independent TLR4 signalling) in the CNS, but immunosuppressive (inhibiting LPS-induced TLR4 signalling) in the peripheral immune system [23]. Morphine can lead to leakage of Gram-negative bacteria and thus LPS from the intestine [25]. As LPS is the major exogenous ligand for TLR4 [24], it might be that in vivo, morphine increases LPS which activates TLR4, as part of an underlying sepsis induced by the opioid [25]. There are likely differences in these wide-ranging immune signalling effects between different opioids. We thus cannot extrapolate effects and findings from one opioid to another opioid or all opioids as a group. Therefore, a study assessing the effect of morphine, as the prototypical opioid, cannot be considered to be the same for other or all opioids [6].

The immune effects of opioids which might influence cancer progression in patients was evaluated in a systematic review. There were five studies which assessed the effect of opioids on anti-tumour immunity in patients with cancer not undergoing surgery [22]. All five studies assessed the immune effects of morphine (no other opioid was evaluated). These studies reported variable effects on different aspects of cancer-related immune function. None of these studies measured the clinical effects of morphine. Thus, the clinical significance of these immune effects is unknown [22].

As well as immune effects, opioids have multiple other effects on mechanisms that regulate cancer development and growth which might impact on survival. These include effects on molecular targets involved in the regulation of cancer proliferation angiogenesis, invasion, and metastasis [17,18,19,20,28]. Opioid receptors are expressed on various cancer cells, including breast, bladder, colon, and lung [29,30]. Thus, opioids can have direct effects on the cancer itself. The molecular effects of morphine in the tumour microenvironment on tumour progression pathways have been reviewed elsewhere in this Special Issue [28].

### MOR Expression and Cancer Progression

The MOR is expressed on cancer and non-cancer cells of the tumour microenvironment [28,30]. Several studies have assessed the effect of MOR expression and polymorphisms with cancer progression. MOR overexpression has been associated with the development of metastasis in patients with prostate, oesophageal and lung cancer [31,32,33]. Hepatocellular carcinomas with high MOR expression were histologically more aggressive and associated with a worse prognosis [34]. In 236 patients with pancreatic adenocarcinoma, high expression of MOR when combined with high opioid doses were associated with poor prognosis [35]. In patients with lung cancer, MOR expression was increased in the cancer compared to adjacent tissue (*p* = 0.024) [32]. There is increased MOR expression within the tumour of patients with metastatic lung cancer compared to those without metastasis (*p* = 0.001) [32]. In patients with metastatic prostate cancer, increased tumour MOR expression, independent of opioid dose, was associated with shorter survival (HR 1.55; *p* < 0.001) [33].

The A118G MOR receptor polymorphism, which confers a reduced receptor response to opioids, was associated with increased cancer-related survival in invasive breast cancer [36]. Chinese people with the A118G MOR receptor polymorphism had a lower incidence of oesophageal cancer [37]. However, this might be cancer-type or population dependent, as North-eastern Polish patients with the A118G allele were more likely to develop breast cancer [38].

Although there might be cancer type and populations differences, increased tumour MOR expression and increased receptor response to opioids has been associated with shorter survival in patients with cancer. Shorter survival seems to be exacerbated by prescribed opioids. These diverse effects of opioids on immunity and other mechanisms that regulate cancer growth might impact on survival. The clinical effects of opioids on survival are reviewed in the following sections.

## 4. Clinical Effects of Opioids on Survival in Patients with Cancer

The effect of opioids on prognosis in patients with cancer not undergoing surgery has been systematically reviewed [11]. In this systematic review, there were studies focusing on patients in the last days to weeks of life and longer-term studies, assessing the effects of opioids in in patients with months to years left to live. In the 13 studies focusing on patients in the last days to weeks of life, there were mixed effects of opioids on survival. Opioid dose was often dichotomised into high and low doses. Some studies showed an association between opioid dose and decreased survival, others showed an association with increased survival and other studies did not find an association. The dichotomised opioid dose, divided patients into two groups depending on the opioid dose they received. The dose cut offs were often very high, in the 100’s of mg OME a day–thus patients in the low opioid dose groups, were also often on very high doses. These mostly retrospective studies were generally methodologically poor. They were mostly very short term studies, usually only including patients with a very limited life expectancy such as from admission to a hospice, or only measured opioids that were taken in the last day(s) of life [11]. From these studies, the highest quality end of life study in this systematic review was a secondary data analysis [39]. This analysis assessed the effects of different opioid doses, based on the daily intravenous morphine-equivalent dose (IVME). It showed that doses equal to or below 17 mg/day (the lowest dose group) were associated with a longer survival compared with doses above 20 mg/day. Patients on ≤17 mg/day IVME had a mean survival of 27 days; compared to 12 days for patients on 20–25 mg/day IVME [39]. These are much lower dose cut offs compared to the other studies and even in this analysis, which primarily characterised patients based on 200 mg/d and 600 mg/d IVME. These are astronomical doses compared to what is commonly used today. Furthermore, in most of these studies only dichotomising opioid dose based on such high doses, the signal of effect of opioids on survival might be lost, as it is only seen in the lowest dose decile in this analysis [39]. All other dose deciles had a mean time until death of between 7 and 15 days, with no dose dependent effect, outside of the aforementioned lowest dose decile [39]. This study also notes that in a hospice population, survival is influenced by complex factors, many of which might not be measurable [39].

In this systematic review there were seven studies in patients with a prognosis of months to years [11]. These studies tended to be larger and of better quality. Six out of the seven studies reported an association between strong systemic opioid use or increasing dose and shorter survival. There were limitations to these studies and none of them assessed causality, only associations. None of these studies had survival as a primary, powered, endpoint. They included different and variable populations, there was also no consistency when opioids were started, the duration of opioid administration and from when survival was measured. The main difficulty with these studies is that the control groups were not directly matched to the opioid group, as they were patients who did not need (or at least did not have) opioids or had lower doses of opioid. The control group was not a matched group of patients who had pain but did not have opioids. Thus, we do not know if it was the opioid causing a decrease in survival or if was that the opioid was needed to control pain in more painful aggressive cancer, which was the cause for the decreased survival. Opioids might be needed at a higher dose to control pain in patients with a more inflammatory (and thus painful) cancer, and it is the more aggressive cancer which decreases survival, rather than the opioid, which is at a higher dose to control pain. Thus, these studies only show associations not causality of the effect of opioids on survival [11].

Since this systematic review, there have been several further studies assessing the association between opioids and survival in patients with cancer. These are briefly overviewed, before being summarised at the end of this section, as they were not included in the above systematic review [11].

In a retrospective tumour registry data study, 1386 patients newly diagnosed with stage IV non-hematologic malignancies were identified. Opioid use within 90 days of commencing oncological treatment was divided into low dose opioids (<5 mg/d OME) and higher dose opioids (≥5 mg/d OME). 762 patients were on low dose opioids and 624 higher dose opioids. The patients on higher opioid doses had a shorter median survival (5.5 vs. 12.4 months; *p* < 0.0001; HR 1.4; *p* < 0.0001) [40].

In 103 patients with metastatic pancreatic adenocarcinoma receiving chemotherapy, patients on low dose opioids (<5 mg OME/d) survived longer than patients on higher dose opioids (≥5 mg OME/d). The median overall survival was 315 vs. 150 days (HR 2.76) [41].

In a Japanese prospective cohort study of 150 patients with advanced non-small cell lung cancer the association between opioids and overall survival was assessed. Median overall survival was 242 days in patients receiving opioids and 627 days those not on opioids (*p* < 0.001). Having any dose of opioid was associated with shorter survival as when opioids were administered any time during the clinical course survival was shorter; this was independent of other factors (HR 1.73; *p* = 0.01). no difference in survival was seen when a 60 mg OME/d cut off was used [42].

In a secondary analysis of an international prospective, longitudinal study of 1739 adults with advanced cancer receiving palliative care, there was a significant association of patients on opioids with reduced survival, vs. those not on opioids (HR 1.59; *p* < 0.001). Increased CRP was associated with reduced survival (*p* < 0.001). There was a weaker association of opioids with survival when adjusted for CRP (HR 1.38; *p* = 0.029). This indicates that although there is an association of opioids with reduced survival, the effect is partly explained by increased inflammation, possibly due to a greater inflammatory response due to the cancer, potentially causing more pain, necessitating opioid use [12].

A single Thai site retrospective study of 317 patients with cancer referred to palliative care assessed opioid dose and survival. Pooling all opioids, the median survival for patients with OME  ≤ 30 was 47 days and OME > 30 was 31 days (*p* = 0.52). In a post hoc subgroup analysis of the 118 patients that had only received morphine, median survival for ≤30 mg/d morphine was 47 days and >30 mg/d morphine was 31 days; >30 mg/d morphine was associated with shorter survival (HR 4.13; *p* < 0.01) [43].

A retrospective cohort study of 203 Chinese patients explored the association of cancer-related pain and opioids on survival in patients with advanced cancer. 106 patients were not on opioids, 46 were on low-dose opioids, and 51 high-dose opioids; the high/low opioid dose cut off was 80 mg/d OME. Patients taking opioids had a shorter cancer-related survival compared to those not taking opioids (HR 2.10; *p* < 0.01); there was no difference between high and low opioid dose groups. Patients not taking opioids mostly had mild pain and thus did not need opioids [44]. The same article also described a systematic review and meta-analysis. 28 studies (all cohort; 27 retrospective) were included. 17 studies reported opioids use in the treatment of cancer-related pain. There were differences between cancer types, with opioids being associated with decreased survival in breast, colorectal, pancreatic and haematological cancers, but not in lung, prostate, and mixed cancers. Overall, the studies combined showed that opioids for cancer-related pain treatment were associated with a decreased survival in patients with cancer. This was stronger for opioids required vs. no opioids (HR 1.53, *p* < 0.001), but remained in the high-dose vs. low dose (80 mg/d OME cut off) opioid comparison (HR 1.05, *p* = 0.002) [44].

A study pooled data from 8 clinical trials including 3441 patients with advanced gastrointestinal cancers; 1277 received an opioid and 2164 did not. 2 trials were in patients with advanced pancreatic cancer, 2 advanced gastric cancer, 1 advanced hepatocellular carcinoma, and 3 trials were in patients with advanced colorectal cancer. Patients with a higher ECOG performance status, and with pancreatic cancer were more likely to be on an opioid (*p* < 0.001). Mean follow-up was 11 months. Opioids were associated with shorter survival in patients with pancreatic (HR 1.25; *p* = 0.007), gastric (HR 1.73; *p* < 0.001), hepatocellular carcinoma (HR 1.84; *p* < 0.001), and colorectal cancer (HR 1.65; *p* < 0.001) [45].

In a database study of 5770 older adult patients with advanced pancreatic cancer in the United States, 29% were prescribed opioids for at least 60 days (refusal, adherence, non-Medicare prescription is not known). Median survival (time from diagnosis to death) was increased in those with opioid prescriptions (6 vs. 4 months; HR 0.80; *p* < 0.0001) [46]. The real strength of this study is the size, however prognostic factors such as performance status, pain, and treatments were not control for. The association of opioid prescription with increased survival in patients with pancreatic cancer goes against other studies in patients with advanced pancreatic cancer, which report an association between opioids and decreased survival [41,44,45].

An editorial linked to this database study [46], emphasises some important points in relation to this type of study and this line of research in general [47]. This includes differences between the intervention and comparator groups, which can be difficult to control for, especially with a database analysis. Opioid prescriptions themselves might mean patients receiving more attentive, personalized care, as there were more palliative care referrals in this group [47]. It also emphasises the limitations which are also relevant to many of the aforementioned studies. Associations with survival might be determined in retrospective studies but causality cannot be inferred. Furthermore, patients on opioids are likely to have more pain, which might be due to a more aggressive cancer which is the primary cause for the worse prognosis [48]. Specifically to this study [46], patients who received a 60-day opioid prescription might select for a population expected to survive long enough to receive such opioid prescriptions [47].

Overall, especially in the better-quality studies, opioids seem to be associated with decreased survival in patients with cancer. Many of these studies indicate that opioids, at any dose, even comparatively low doses (with a cut off of >5 mg OME/d) might decrease survival, compared to no opioids or very low opioid doses. When the comparator group includes people on moderate to high doses of opioids any potential effect of the “high” dose opioid group might be removed as any effect will also be seen in the comparator “low” dose group, as the low dose comparison group includes people on relatively high doses. This lays doubt to the methodology of the high opioid-dose cut offs used in some studies, as the high opioid dose cut off might miss an effect of low dose opioids, as the low dose group will be contaminated with patients on relatively high doses of opioids. There are many other limitations to these studies [11,47]. These studies are assessing associations, not causation. The opioid-reduced survival association is influenced by many factors, not all of which can be adjusted for in the multivariate adjustments to the HR being made. This is paramount as the comparator group is not matched to the opioid group, as they often have less severe pain. Opioids are most commonly prescribed for pain in patients with cancer [1,2,49]. Pain itself is often considered to influence survival in patients with cancer; this is overviewed in the next section.

## 5. Pain and Survival in Patients with Cancer

Pain control is essential for many reasons [10]. The association of pain and survival in patients with cancer will be briefly reviewed to help put into context the main use of opioids in patients with cancer. There is a theoretical benefit that when pain is controlled, there might be an improvement in overall survival in advanced colorectal cancer patients. Mechanisms include that pain might be immune suppressive per se, if supressing the anti-tumour aspects of the immune system this might lead to increased cancer growth [50], pain might also increase endogenous opioid release, increasing MOR activation suppressing immune function. Furthermore, as discussed above, opioids prescribed for pain, increase MOR activation, which is often associated with decreased survival.

A systematic review included 50 (mostly observational) studies assessing the effect of pain on survival in patients with cancer. In most studies of patients with breast, colorectal, and lung cancer, pain severity was not associated with length of survival. In advanced prostate cancer, 11 out of the 17 included studies reported an association between pain (in 5 studies analgesic use was used as a surrogate for pain) and decreased survival. It is thus unclear the relative importance of pain or opioids for pain control in the associated decreased survival [51].

Subsequent to this review, in 150 patients with incurable non-small cell lung cancer, there was no relationship between pain severity at diagnosis and length of survival [42]. In 103 patients with metastatic pancreatic adenocarcinoma receiving chemotherapy, baseline pain was not associated with length of survival [41].

A retrospective cohort study of 203 Chinese patients explored the association of cancer-related pain and opioids on survival in patients with advanced cancer. Pain was divided into low, moderate and severe. Severe pain was associated with shorter survival (HR 4.38; *p* < 0.001). However, the pain severity was higher in patients taking opioids [44]. Clinical outcome data from a chemotherapy clinical trial of 569 patients with advanced pancreatic cancer showed less severe pain at baseline was associated with better overall survival (*p* = 0.01) [52].

**Figure 2:**
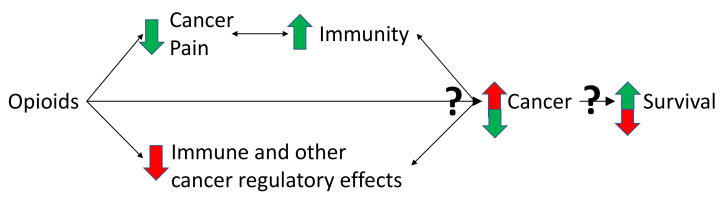
**Triangulation of the effects of opioids on pain, immunity and cancer.** Cancer can cause pain, by nociceptive, neuropathic, and inflammatory mechanisms, partly caused by the immune response to cancer [1,2,53]. It is this pain state that necessitates opioid use [1,2,49]. Pain is potentially immunosuppressive which might worsen cancer outcomes in some cancer types [44,51,52]. By reducing pain, opioids might have beneficial effects on immune function, the cancer and potentially survival [44,51,52]. However, some opioids suppress immune function, which might decrease anti-tumour immunity and promote cancer growth [6]. Furthermore, there are non-immune effects of opioids on cancer cell regulation [17,18,19,20,28]. Opioids can also act directly on the MOR on cancer and non-cancer cells of the tumour microenvironment [28,30]. Together, these multiple effects converge to influence cancer growth and survival [11,12,54]. The balance of these effects is critical and might be dependent on the immune properties of the opioid used and the cancer type [6,15]. Many of the aforementioned effects are bidirectional (depicted by double arrowed lines in the figure). The immune system, via microglia and cytokines, influences the pain state [55]. Activated immune cells can also produce endogenous opioids, as well as morphine [56]. The immune system and the cancer are constantly influencing each other, with processes such as immunoediting and immunosculpting [57]. The aforementioned interactions lead to either cancer cell destruction or growth. How the cancer progresses influences survival. Green arrows depict a beneficial effect, red arrows depict a detrimental effect of opioids on the immune system, cancer development and survival. Question marks are used to highlight uncertainly of the net balance of effects, which might vary depending on the opioid. Adapted with permission from Boland, J.W. et al. *Br. J. Pharmacol.* **2018**, *175*, 2726–2736. [15]; Boland, J.W. et al. *Br. J. Cancer* **2014**, *111*, 866–873 [22]; *Eur. J. Clin. Pharmacol*. **2020**, *76*, 393–402. [12].

Some studies report an association between pain and decreased survival and several potential mechanisms have been postulated. Based on the current literature the association between pain and survival seems dependent on the underlying cancer. Prospective studies are needed to better understand how pain and opioids might influence survival in patients with different cancers [51]. The interactions of opioids on pain, immunity and cancer are shown and described in Figure 2. It must be noted that the detriment of poor pain control in patients with cancer goes far beyond any theoretical or real effect on survival.

## 6. Effect of Opioids on Survival in Non-Cancer Populations

Although this article is focused on the effects of opioids on survival in patients with cancer. There are several other areas in which the effects of opioids on survival have been studied. These include studies in chronic non-cancer pain and in shortness of breath in patients with chronic obstructive pulmonary disease. In patients with cancer there is a dynamic relationship between the cancer and immune system, and the immune activation status will likely be different compared to patients in these non-cancer studies. Thus, the findings of these studies are not directly applicable to patients with cancer.

In chronic non-cancer pain, a systematic review compared mortality associated with opioid use compared to non-opioid analgesics. 4 propensity score matched observational studies, with 120,186 patients were included in the meta-analysis. Opioids were associated with an increase in mortality (HR 1.69) [58]. Subsequent to this, in two population-based cohort studies by the same research group, the effect of opioids on cancer development and survival was assessed [59,60]. The cancer development study included 63,610 Taiwanese patients (50,888 on opioids) with chronic pain. There was an increased risk of cancer development with long-term opioid use compared with no opioids in patients with chronic pain (HR 2.66; *p* < 0.001) [59]. In 1716 patients (286 on opioids), those receiving long-term opioids for chronic pain before cancer diagnosis had a shorter survival once they developed cancer (HR 3.53; *p* < 0.001) [60]. These studies show an association between opioid in patients with chronic pain and cancer development and decreased survival in those who develop cancer. There is also an association between opioids and decreased survival in those with non-cancer chronic pain.

In chronic obstructive pulmonary disease, a Swedish population-based cohort study, of 2249 patients starting long term oxygen therapy for severe shortness of breath there was a dose response relationship of opioids with mortality. >30 mg/d OME was associated with increased mortality (HR 1.21); ≤30 mg/d OME was not [14]. In a retrospective population-based cohort study of patients with chronic obstructive pulmonary disease, those prescribed opioids were more likely to attend the emergency department, have pneumonia and die within 30 days of starting opioids. All-cause mortality was increased in patients on opioids (HR 1.76; *p* < 0.0001) as was chronic obstructive pulmonary disease or pneumonia-related mortality (HR 2.16; *p* < 0.0001) [61]. Association (not causation) must be emphasised as it is possible that worsening illness necessitated opioid use for symptom control.

In these non-cancer populations, there is an association between opioids and decreased survival, although these give an interesting comparison, people included in these studies are different to the population of interest in this article and thus extrapolation of opioid effect is not possible.

## 7. Future Work

Prospective studies need to ascertain causality and differentiate between opioids, pain and cancer-mediated effects on survival in patients with different cancer types. There might also be differences between the effects of opioids on survival between different cancers.

Studies must also enable the determination of an effect of opioid doses, with the understanding that very low doses of opioids might also affect survival. Based on limited clinical data (as most patients are on morphine and opioids often pooled together), there seem to be differences between opioids which need to be elucidated in future studies. Studies are also needed in patients with cancer who are living longer, to understand the long-term effect of opioids of patients living with long term cancer [1]. Another line of future work needs to be effect of survival of opioids for pain management in the context of immunotherapies to treat cancer [62].

## 8. Conclusions

There have been many studies assessing the effect of opioids on survival in patients with cancer pain. These have methodological limitations and are assessing association not causation. Most of the better-quality clinical studies report an association between long term opioid use and reduced survival in patients with cancer; causality cannot be inferred. Many of the studies pool different cancers, but when separated into specific cancer type, all have some studies showing decrease survival associated with opioids. Similar findings of reduced survival associated with opioids are echoed in non-cancer populations.

There are many biases, including the control group not needing opioids. Thus, the group of patients not having opioids might have less severe pain and potentially a less inflammatory and less aggressive cancer. Patients with more painful progressive inflammatory cancers need more opioids and the shorter survival might be mediated by the underlying cancer itself. Furthermore, pain potentially has effects of its own on immunity and possibly survival. These effects of opioids and pain are not in isolation. The interactions of opioids on pain, immunity and cancer are shown and described in Figure 2. To understand the complexity of the association of opioids with decreased survival, the relationships between the underlying cancer, opioids, pain and survival need further study.

It would be difficult to do a study where the control group (eg patients with cancer in pain not receiving opioids) is matched to the opioid group, to monitor the effects of opioids on survival. So the methodology used in the study of opioids and survival give useful associations but cannot look at causation. Therefore, these studies are not definitive in assessing the effects of opioids on survival. The results of some of the studies can be quite stark showing a marked decrease in survival in patients receiving opioids. However, in the clinical context this must be conveyed with caution to patients and their families as results do not take into account potential differences in tumour aggressiveness which has potential to cause pain. Furthermore, pain itself maybe immunosuppressive and thus not treating the pain not only can have profound effects on quality of life but might also negatively impact on immunity and potentially survival as well (good clinical data to support this).

In terms of care in the last days of life, many studies are methodologically flawed and there are no high-quality data. No consistent effect of opioids on survival is reported in this cohort. Furthermore, comfort is a priority and must outweigh any hypothetical concerns in patients in the last days of life. Further research into causality is needed with clinical outcomes in patients with different cancer types needing opioids with a prognosis of months to years. There will continue to be a need for good pain control in patients with cancer which will involve optimisation of non-pharmacological and other interventions, non-opioid analgesics as well as judicious consideration of opioid. Based on limited clinical data, there seem to be differences between opioids.

## Figures and Tables

**Table 1:** Effects of opioids on different organ systems.

System	Effects
Gastrointestinal	Constipation, xerostomia, nausea, vomiting, delayed gastric emptying, gastro-oesophageal reflux, constriction of the sphincter of Oddi
Neurological	Analgesia, delirium, hallucinations, sedation, myoclonus, hyperalgesia, seizures, headaches, euphoria, dysphoria, tolerance, dependency
Cardiovascular	Bradycardia, hypotension
Pulmonary	Respiratory depression, decreased cough reflex, non-cardiogenic pulmonary oedema
Urological	Urine retention, decreased urine production, altered renal function
Endocrinological	Hypogonadism, sexual dysfunction, osteoporosis
Immunological	Decreased neutrophil, macrophage, natural killer cell, T cell function and cytokine dysregulation

Adapted with permission from Boland, J.W. *Clin. Med.* **2013**, *13*, 149–151 [9].
